# Multi-Level Social Capital and Subjective Wellbeing Among the Elderly: Understanding the Effect of Family, Workplace, Community, and Society Social Capital

**DOI:** 10.3389/fpubh.2022.772601

**Published:** 2022-04-15

**Authors:** Zongyou Xu, Wenjie Zhang, Xuewen Zhang, Yixi Wang, Qing Chen, Bo Gao, Ningxiu Li

**Affiliations:** ^1^Department of Health Related Social and Behavioral Science, West China School of Public Health and West China Fourth Hospital, Sichuan University, Chengdu, China; ^2^Medical School, Hubei Minzu University, Enshi, China; ^3^Department of Academic Affairs, West China School of Medicine and West China Hospital, Sichuan University, Chengdu, China; ^4^School of Integrated Traditional Chinese and Western Medicine, Jining Medical University, Jining, China

**Keywords:** subjective wellbeing, social capital, family, workplace, community, society, aging, China

## Abstract

**Background:**

Maintaining the subjective wellbeing of the elderly people is one of the major concerns in promoting health aging. This study concerned the influence of multi-level social capital on subjective welling and explored the affecting path among the elderly.

**Methods:**

A total of 1,078 elderly individuals anonymously and effectively surveyed in 2018, data was collected including their family, workplace, community, society social capital and subjective wellbeing, we used the structural equation modeling to test the hypothesis relationships among the variables.

**Results:**

We found that the total score of subjective wellbeing among the aging participants was 72.36 ± 10.08 on a range of 0–100. Family (β = 0.151, *P* < 0.001), workplace (β = 0.090, *P* < 0.001), community (β = 0.163, *P* < 0.001) social capital had a direct positive effect on subjective wellbeing. Society social capital had a direct positive effect on family (β = 0.253, *P* < 0.001), workplace (β = 0.585, *P* < 0.001), community (β = 0.438, *P* < 0.001) social capital. And society social capital had an indirect positive effect on subjective wellbeing through the mediating role of family, workplace, and community social capital.

**Conclusion:**

The research demonstrated that all the micro, meso and macro levels of social capital have protective effects for subjective wellbeing through direct or indirect way, inspiring to provide continuous improvement measures for multi-level social capital aimed at the elderly people.

## Introduction

Subjective wellbeing encompasses both cognitive and affective aspects which reveal the assessment of overall life, the presence of positive emotions and the absence of depression (1). The reason for focusing on the elderly is that subjective wellbeing is intimately associated with age and health. In one respect, physical illness with increasing age is the determinant of impaired subjective wellbeing. On the other hand, subjective wellbeing might play a more important role in reducing the risk of chronic physical illness among the elderly people (2). Evidence is accumulating that subjective wellbeing is associated with many positive outcomes including healthy aging, as high subjective wellbeing such as optimism, life satisfaction and positive thoughts is always able to cause better health and longevity (3). As the second largest global economy, China has entered an accelerating period of the aging population. In 2050, China will be one of the countries in the world with the highest percentage of aged people with a prolonged life expectancy (4), it becomes one of the key societal aspirations to improve the subjective wellbeing of someone elderly.

Researches have shown that social capital is one of the factors affecting the subjective wellbeing of the elderly. A study made in six low and middle income countries suggested that improving the social capital of older adults with chronic diseases could potentially improve their subjective wellbeing, the measure of social capital focuses specifically on social participation (5). Pirkko et al. revealed that interventions of empowering the elderly, promoting peer support and social integration can improve wellbeing for those lonely, older people (6). A review showed that transition to retirement age, elderly people maintain the health and wellbeing by accepting the interventions of offering an explicit social role with group support, such as active volunteering, as the social contact increasing in helping others (7).

There has been much evidence about the reason why issues relating to social capital is particularly relevance to the subjective wellbeing for elderly groups. First, because of health deterioration, dies of spouses and partners, the elderly are more likely to feel lonely and isolated with fewer confidential relations (8). Second, the shrinking social networks and limited social contact in the aging process could make older adults producing negative assessment or perceptions about their performance in family, community, or the larger society, the self-perceived uselessness normally impact one's physical and mental wellbeing (9). Moreover, both formal contacts such as workplace and informal contacts such as immediate family was found essential for mental wellbeing among old adults (10).

As a concept that has been applied in many fields, social capital has been defined in different ways, there is more debate about the measurement methods. Bourdieu defines social capital as a collection of actual or potential resources embedded in the social structure or interpersonal network that can be controlled and utilized by individual actors or organizations, by utilizing the individual actors or organizational purpose (11). Coleman explains social capital from a perspective of function, believing that such structural resources are capital property owned by individuals characterized by social structural resources (12). Putnam describes social capital as the characteristic of social organizations, including trust, norms, and citizen participation network, which could improve the efficiency of society by promoting coordination and promoting the behavior of citizen participation in cooperation (13). Overall, there are conflicts created by different definitions because the conflicting purpose and service scope have included in social capital. Robison preferred defined social capital as the sympathy or empathy of one person or group for another person or group (14), and this sympathy or empathy produces at least five distinct motivations, including consumption, self-respect, belonging, good will and sharing which could influence people's behavior (15). We placed social capital within the aging group and explored what social relationships that they can derive from other people or groups, obtaining the potential benefits, advantages, and preferential treatment acting on subjective wellbeing. This social relationship may come from many groups and sources, although most studies only explore the relationship between subjective wellbeing and the single direction and level of social relationship.

**Figure 1:**
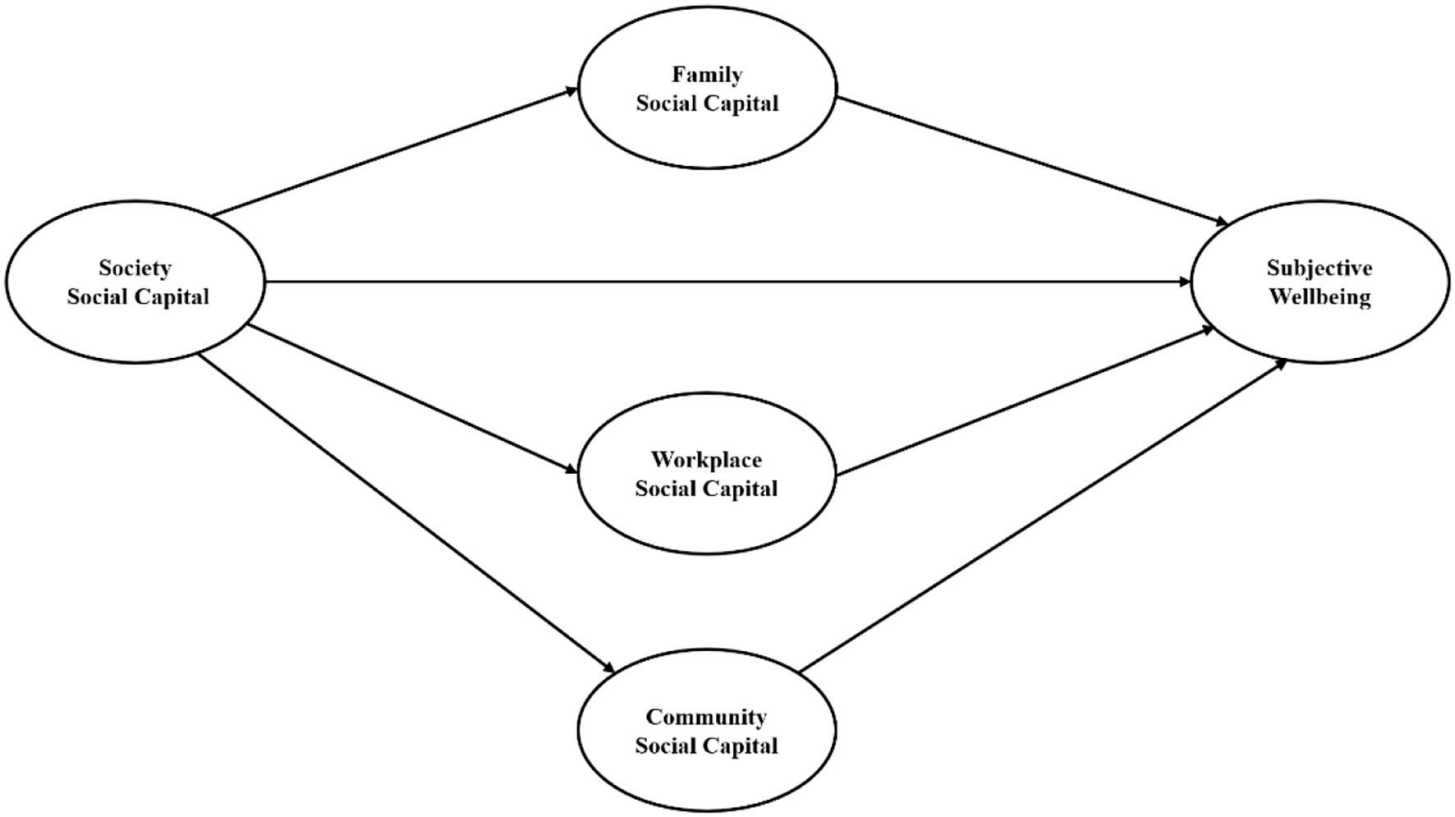
The hypothesized model.

In this study, we interested in conducting a more systematic social capital framework covering various aspects of resources that individual obtains from social networks to maintain wellbeing. The aim of this article is therefore to focus on the relationship between subjective wellbeing with micro (family), meso (workplace, community), and macro (society) levels of social capital. Based on the literatures, the following assumptions were presented: (1) family social capital has an direct effect on subjective wellbeing, (2) workplace social capital has an direct effect on subjective wellbeing, (3) community social capital has an direct effect on subjective wellbeing, (4) society social capital has an direct effect on subjective wellbeing, (5) society social capital has an indirect effect on subjective wellbeing through the mediating role of family, workplace, community social capital. The theoretical model was shown in Figure 1.

## Materials and Methods

### Setting and Participants

Data used in this study are obtained from a cross-sectional study made in Chengdu in 2018. As the provincial capital of Sichuan Province in China, Chengdu's aging population level is 21.35% in 2018, far exceeding the national average (17.9%). A multistage stratified random sampling survey was used to acquire the sample. Considering the economic performance and the distribution of aging population, we randomly selected two districts from two city circles of Chengdu, respectively, in the first stage. Next, three communities were randomly sampled from each selected district. In the third stage, we used systematic random sampling to choose qualified elderly people in the chosen communities. Since the retirement age of women is 55 years old in China, residents aged ≥55 years, lived in the chosen communities for no <6 months, no cognitive impairment or diagnosis of dementia matched the inclusion criteria. With an informed consent, participants received face-to-face interviews by trained investigators and completed the questionnaire anonymously. There were 1,078 elderly individuals participated the survey. The ethics of this survey was approved by the Ethics Committee of the West China School of Public Health, Sichuan University.

### Measures

The questionnaire was designed by an expert panel conducted in one National Natural Science Fund Project (71603176) in China. The questionnaire included six parts, demographic characteristics (gender, age, marital status, education attainment, employ status, chronic disease, 2-week prevalence, hospitalized within a year, smoking, and drinking), family social capital, workplace social capital, community social capital, society social capital and subjective wellbeing. The social capital index for the elderly has been constructed and verified widely. First, we constructed an index system framework with literature review and panel discussion and then evaluated and screened the indicators of the system with two rounds of Delphi consultation among 34 experts. The weights of the indicators were determined with analytic hierarchy process. The established index system is applicable for the measurement and evaluation of social capital for aging population.

#### Family Social Capital

The questionnaire of family social capital comprised 3 dimensions: family size (2 items), family relation (3 items) and family support (2 items). Five-point Likert scale ranging from 1 (highly disagree) to 5 (highly agree) was being used for evaluating, the higher score revealed the higher level of family social capital. The Cronbach's Alpha coefficients of this questionnaire was 0.480.

#### Workplace Social Capital

The questionnaire of workplace social capital comprised 4 dimensions: workplace participation (4 items), workplace support (2 items), workplace trust (2 items) and workplace belonging (2 items). Five-point Likert scale ranging from 1 (highly disagree) to 5 (highly agree) was being used for evaluating, the higher score revealed the higher level of workplace social capital. The Cronbach's Alpha coefficients of this questionnaire was 0.595.

#### Community Social Capital

The questionnaire of community social capital comprised 5 dimensions: community participation (5 items), community support (1 items), community belonging (3 items), community cohesion (1 items), community trust and safety (3 items). Five-point Likert scale ranging from 1 (highly disagree) to 5 (highly agree) was being used for evaluating, the higher score revealed the higher level of community capital. The Cronbach's Alpha coefficients of this questionnaire was 0.918.

#### Society Social Capital

The questionnaire of society social capital comprised 7 dimensions: Employment and labor-related security system (2 items), pension security (1 items), medical insurance (2 items), unemployment insurance (1 items), health resource allocation (4 items), society trust (1 items) and social equality (2 items). Five-point Likert scale ranging from 1 (highly disagree) to 5 (highly agree) was being used for evaluating, the higher score revealed the higher level of society social capital. The Cronbach's Alpha coefficients of this questionnaire was 0.322.

#### Subjective Wellbeing

The WHO-5 is one of the most widely used scales for assessing subjective wellbeing which comprises 5 items, measuring the feeling of participants over the last two weeks (16). (1) I have felt calm and relaxed, (2) I have felt cheerful and in good spirits, (3) I have felt active and vigorous, (4) I woke up feeling fresh and rested, (5) my daily life has been filled with things that interest me. Six-point Likert scale ranging from 0 (at no time) to 5 (all the time) was being used for evaluating, the raw score converts to a range from 0 to 100, higher score revealed the higher level of subjective wellbeing. The Cronbach's Alpha coefficients of this questionnaire was 0.729.

### Statistical Analysis

First, we used descriptive analysis of participant characteristics, family, workplace, community, society social capital and subjective wellbeing. Means and standard deviations (SD) were used for continuous variables, frequencies and percentage were used for categorical variables. Next, we used linear regression analysis to estimate the influence of family, workplace, community, society social capital on subjective wellbeing after adjusting for demographic factors. Finally, structural equation modeling was employed to test hypothesized relationships among multi-level social capital and job satisfaction. The fit between the current data and hypothesized model was assessed through several indicators, adjust goodness of fit index (AGFI), a goodness of fit index (GFI), comparative fit index (CFI), incremental (IFI), and Tucker-Lewis index (TLI) of 0.90 or above, a root mean squared error of approximation (RMSEA) <0.08, indicated an acceptable model fit.

## Results

### Subjective Wellbeing Among Participants

Table 1 shows the demographic characteristics, health status, health behaviors, social capital, and subjective wellbeing of 1,078 participants. The largest proportion of respondents (42.5%) was in the 65–74 age group. Most of respondents were female (67.9%), married (77.5%), graduated from primary school or be illiterate (55.5%), unemployment (56.2%). In terms of health status, about half of them have chronic disease (52.1%), sicked in 2 weeks (47.9%), 19.7% hospitalized last year. In terms of health behaviors, most respondents were never smoking (80.7%), not drinking (87.6%). In terms of the four levels social capital and subjective wellbeing, the average scores of family, workplace, community, and society social capital were 68.09 ± 13.56, 16.27 ± 27.78, 51.93 ± 14.77, 38.47 ± 7.06, respectively. The average scores of subjective wellbeing were 72.36 ± 10.08. Subjective wellbeing was influenced by gender, marital status, education attainment, employ status, chronic disease, 2-week prevalence, hospitalized within a year, drinking.

**Table 1:** Subjective wellbeing among survey elderly participants (*n* = 1,078).

**Variables**	***n* (%)**	**Mean ±SD (Range)**	***P* (Maximum)**
**Gender**			0.015^*^
Male	346 (32.1)	73.77, 9.28	
Female	732 (67.9)	71.70, 10.38	
**Age (years)**			0.060
55~64	393 (36.5)	73.20, 8.83	
65~74	458 (42.5)	72.20, 10.67	
75~	227 (21.1)	71.24, 10.81	
**Marital status**			0.001^*^
Married	835 (77.5)	72.98, 9.42	
Unmarried, divorced, or widowed	243(22.5)	70.26, 11.87	
**Education attainment**			<0.001
Primary school or below	598 (55.5)	70.80, 11.04	
Junior high school	284 (26.3)	73.96, 8.66	
Senior school	133 (12.3)	75.01, 7.60	
Junior college or above	63 (5.8)	74.41, 8.52	
**Employ status**			<0.001^*^
Employed	41 (3.8)	75.32, 6.25	
Retired	425 (39.4)	74.32, 8.65	
Lose the job	6 (0.6)	72.67, 8.91	
Unemployment	606 (56.2)	70.79, 10.93	
**Chronic disease**			<0.001^*^
Yes	562 (52.1)	70.96, 11.19	
No	516 (47.9)	73.89, 8.48	
**Two-week prevalence**			<0.001^*^
Yes	516 (47.9)	70.57, 11.62	
No	562 (52.1)	74.01, 8.10	
**Hospitalized within a year**			0.001^*^
Yes	212 (19.7)	69.92, 11.42	
No	866 (80.3)	72.96, 9.64	
**Smoking**			0.796
Yes	134 (12.4)	72.90, 11.14	
Quit	74 (6.9)	72.49, 8.78	
Never	870 (80.7)	72.27, 10.03	
**Drinking**			<0.001^*^
Yes	134 (12.4)	76.06, 6.07	
No	944 (87.6)	71.84, 10.43	
**Family social capital**		68.09, 13.56	100
**Workplace social capital**		16.27, 27.78	100
**Community social capital**		51.93, 14.77	100
**Society social capital**		38.47, 7.06	100
**Subjective wellbeing**		72.36, 10.08	100

* *P < 0.05*.

### Association Between Social Capital and Subjective Wellbeing

Table 2 shows the association between social capital and subjective wellbeing. In model 1, the results revealed that family social capital, workplace social capital, community social capital were significantly correlated with subjective wellbeing (*P* < 0.05). In model 2, the results showed that the three level social capitals were still significantly correlated with subjective wellbeing (*P* < 0.05) adjusting for demographic characteristics including gender, age, marital status, education and employ status, graduating from junior high school were significantly correlated with subjective wellbeing (*P* < 0.05). The results were same again in the model 3 after adjusting for smoking and drinking, the drinking elderly people had a higher subjective wellbeing (*P* < 0.05).

**Table 2:** Linear regression analysis of association between social capital and subjective wellbeing.

	**Model1**	**Model2**	**Model3**
	**β (95%)**	**β (95%)**	**β (95%)**
Family social capital	0.14 (0.06, 0.15)*	0.14 (0.05, 0.15)*	0.14 (0.05, 0.15)*
Workplace social capital	0.05 (0.03, 0.07)*	0.11 (0.01, 0.07)*	0.11 (0.01, 0.07)*
Community social capital	0.10 (0.03, 0.11)*	0.08 (0.01, 0.10)*	0.08 (0.01, 0.09)*
Society social capital	0.03 (−0.04, 0.13)	0.02 (−0.06, 0.12)	0.01 (−0.07, 0.10)
**Gender (reference: male)**		1	1
Female		−0.05 (−2.35, 0.27)	−0.04 (−2.31, 0.77)
**Age (Reference:55** **~** **)**		1	1
65~		0.01 (1.32, 1.46)	0.03 (−0.74, 2.03)
75~		−0.02 (−2.19, 1.30)	0.03 (−1.14, 2.38)
**Marital status (reference: single)**		1	1
Married		−0.01 (−1.97, 1.42)	−0.01 (−1.90, 1.43)
**Education (reference: primary school or below)**		1	1
Junior high school		0.07 (0.13, 3.12)*	0.06 (-0.05, 2.89)
Senior school		0.05 (−0.57, 3.59)	0.05 (-0.66, 3.44)
Junior college or above		0.02 (−1.92, 3.44)	0.01 (-2.20, 3.08)
**Employ status (reference: employed)**		1	1
Retired		−0.01 (−3.45,3.03)	0.02 (−2.86, 3.52)
Lose the job		−0.01 (−9.42, 7.69)	−0.01 (−8.52, 8.30)
Unemployment		−0.04 (−4.25, 2.73)	−0.02 (−3.90, 2.98)
**Chronic disease (reference: yes)**			1
No			0.05 (−0.77, 2.66)
**Two-week prevalence (reference: yes)**			1
No			0.11 (0.52, 3.94)*
**Hospitalized within a year (reference: yes)**			1
No			0.07 (0.21, 3.16)*
**Smoking (yes)**			1
Quit			0.03 (−1.75, 3.79)
Never			0.05 (−0.64, 3.41)
**Drinking (yes)**			1
No			–0.10 (–4.94, –1.13)*

* *p < 0.05 Model 1: adjusted for demographic characteristics including gender, age, marital status, education and employ status. Model 2: added for health status including chronic disease, 2-week prevalence, hospitalized within a year. Model 3: added for health behaviors including smoking and drinking*.

### The Effect of Multi-Level Social Capital on Subjective Wellbeing in Structural Model

In the process of correcting the structural equation model, the model adjustment can be made based on the results of the initial model by deleting or adjusting some paths to make the model more identifiable and of realistic significance. According to the path coefficient of the initial model, there was no significant direct effect between society social capital and subjective wellbeing. Therefore, we got the modified model after deleting this direct path in the initial model. The overall model fit indices of the modified hypothetical model were AGFI = 0.901, CFI = 0.919, IFI = 0.920, TLI = 0.908, GFI = 0.921, RMSEA = 0.056, all adaptation indicators meet the model fitting criteria, indicating the final model is suitable and draw up to match effective.

**Figure 2:**
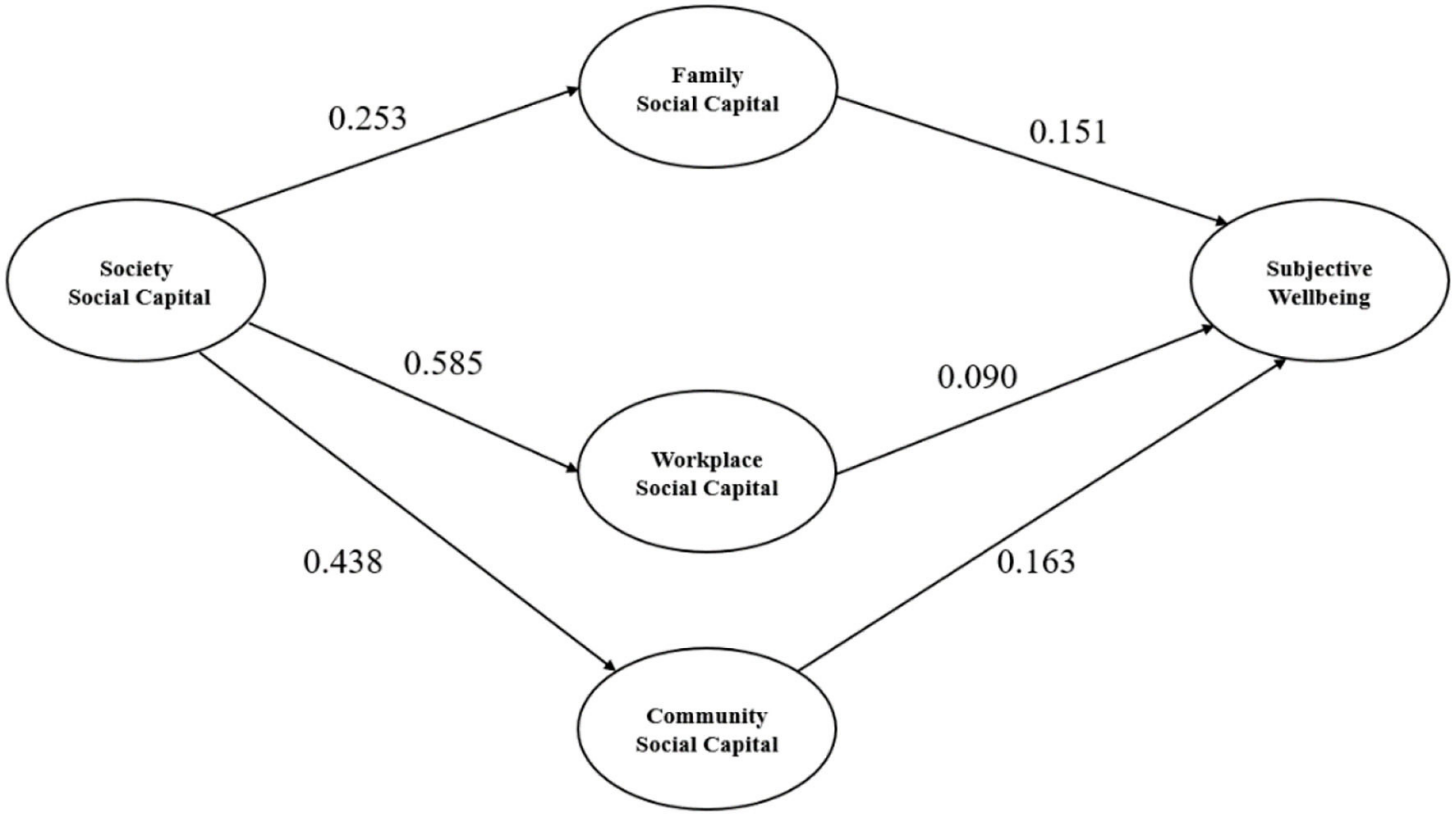
The final model and standardized model paths.

The results of the final model are shown in Table 3 and Figure 2. In terms of direct effect, family social capital had a direct effect of subjective wellbeing (β = 0.151, *P* < 0.001), workplace social capital had a direct effect of subjective wellbeing (β = 0.090, *P* < 0.001), community social capital had a direct effect of subjective wellbeing (β = 0.163, *P* < 0.001), society social capital had a direct effect of family social capital (β = 0.253, *P* < 0.001), society social capital had a direct effect of workplace social capital (β = 0.585, *P* < 0.001), society social capital had a direct effect of community social capital (β = 0.438, *P* < 0.001). In terms of indirect effect, society social capital has an indirect effect of subjective wellbeing through family (β = 0.038, *P* < 0.001), workplace (β = 0.053, *P* < 0.001), and community social capital (β = 0.071, *P* < 0.001).

**Table 3:** The path coefficients between social capital and subjective wellbeing.

**Model pathways**	**Estimated**	**95%CI**
**Direct effects**
Subjective wellbeing ← Family social capital	0.151	0.059–0.256
Subjective wellbeing ← Workplace social capital	0.090	0.033–0.138
Subjective wellbeing ← Community social capital	0.163	0.094–0.231
Family social capital ← Society social capital	0.253	0.168–0.338
Workplace social capital ← Society social capital	0.585	0.523–0.651
Community social capital ← Society social capital	0.438	0.337–0.532
**Indirect effects**
Subjective wellbeing ← Family social capital ← Society social capital	0.038	0.002–0.015
Subjective wellbeing ← Workplace social capital ← Society social capital	0.053	0.003–0.021
Subjective wellbeing ← Community social capital ← Society social capital	0.071	0.005–0.030

## Discussion

This study focuses on the subjective wellbeing among elderly people and explore the effecting path of family social capital, workplace social capital, community social capital, society social capital on subjective wellbeing. The value of this research is incorporate social capital at micro, meso and macro level into the structure, and revealed the mediating path between macro social capital and subjective wellbeing.

The mean subjective wellbeing in this study was 72.36 ± 10.08 which consistent with the general population (17) and represented a good condition of mental health for elderly participants (18). From the aspects of demographic characteristics, the aged people who getting sick in the last 2 weeks or hospitalized within 1 year have a poor subject wellbeing, previous studies have widely demonstrated the relationship between physical illness with subjective wellbeing (19). All hypothesis verified except assuming 4, the results revealed community social capital and society social capital are stronger predictors in all significantly meaningful path.

Family social capital, the representative of micro level, was shown to be directly act on subjective wellbeing among elderly people. From the composition of the family social capital in our study, the quantity and quality of the family relationship are equally important (20). When we define the extension, density and centrality of family network structure, it was essential to take account of the number of members in the network, the number of connections between members and the number of connections within members for which the elderly is an intermediary (21). Meanwhile, the quality of family relationships reflected in extremely close connections and support that old individuals accepted from spouses, children at home, close relatives, and other family members (22, 23). A study made in China found parents aged over 60 having tight-knit family patterns tended to have higher wellbeing compared with highly ambivalent pattern (24). Katie et al. fount that being with spouse leads to better wellbeing and less stress for married couples retired (25). Zhou et al. revealed that living with family is associated with enhancing wellbeing than those living alone. The close connections got from family can be simple interactions such as eating dinner, going for walk, working on a project (26, 27), or practical help such as talking about anxiety with specific family members, receiving financial support and enjoy the warmth of kinship (28).

In this study, Workplace social capital was demonstrated a direct effect on subjective wellbeing, although most older participants are retired or unemployed. Previous studies explored the effects of workplace social capital on mental health, while less attention has been paid to examined the relationship between workplace social capital with subjective wellbeing of the elderly (29, 30). On the one hand, workplace social capital buffers work stress and regulates subjective wellbeing (31). On the other hand, social networks with colleagues in the workplace can provide resources for workers, including health resources (32). For those aging people who have retired from work, their communication with former colleagues has never stopped, and some trade unions in the normative workplaces provide more activities and communication for them. A study made in Europeans found that current or former colleagues, though not as closely as family members, are usually in more frequent contact for the elderly (33). As a result, current or former colleagues form an important part of their confidante network, which is considered as a kind of social network that provides more resources. Jonsson et al. found that about 75% of old individuals would like to continue working after retirement, and that most of these people cited social communication with colleagues as the most important factors other than the economy (34). The workplace social capital of elderly participants in this study is in a poor condition, which does not affect the important role of workplace social capital in promoting subjective wellbeing. If the workplace gives people a sense of belonging and gives them more opportunities to participate in collective activities whether it before and after retirement, they will be less lonely and helpless.

Community social capital in meso level also had a direct positive effect on subjective wellbeing which is consistent with other researches. A study made in Holland found community cohesion, belonging and changes predicts the wellbeing of independently living older adults (35). Choi et al. found that perceived community safety influence wellbeing of older adults, and social cohesion buffers the adverse effects from unsafe neighborhood especially for those physically impaired respondents (36). There are three ways to link community social capital with subjective wellbeing (37), collective effectiveness is the ability of collective action among community members (38), such as recruiting a team of elderly resident volunteers to handle community affairs which could promoting the social participation of them (39). Informal social control refers to the ability of community to prevent uncivilized behavior and maintain safety, older residents are more willing to go out for social activities if they trust the neighbors and neighborhood committees (40). And social contagion is the process in which resources such as public information and mutual aid norms spread faster in communities (41). Community is the most important and the high frequency life scene for most elderly individuals, it is vitally important to building the wellbeing-supportive neighborhoods for older adults (42).

The most important finding of this study is to explore the direct effect of society social capital on subjective wellbeing is not significant, while family, workplace and community social capital mediated the role path. In contrast to social capital at the micro and meso level, the macro level social capital especially for older people has received less attention, despite its relevance in subjective wellbeing (43). Society social capital in this study contains the availability and accessibility to essential employment, pension, medical service, the confidence of public facilities, and trust in social equity. According to the results, less social capital was obtained from the macro level by aging participants. The perceived poor social equity means great differences and negative comparisons among people, especially for those at a disadvantage. Victims of negative social comparisons can evoke a strong sense of relative deprivation, leading to low subjective wellbeing (44). After controlling income, age and education, the increasing perceived fairness of social security and income distribution policies is positively related with subjective wellbeing (45). However, studies have also found that only trust is the major social capital driver affecting wellbeing, and any other form and normative related activities and effective sanctions for macro level show only a relatively small connection to the subjective wellbeing perceived by individuals (46). In this study, the link is manifested as the direct effect between macro social capital and wellbeing is not significant and needs to be driven by micro and meso social capital. It is interesting that social capital will not depreciate with the use of physical assets. On the contrary, the more its stock, the more it depends on (47). Therefore, our study may reveal the dependence of macro social capital of the elderly on the micro and moderate capital stock. Previous studies have explored the interact relationship between family, workplace and community social capital and the mental health and wellbeing of the elderly (48, 49), this paper is the first time to use micro and meso social capital as the mediating variable of macro social capital and subjective wellbeing.

Overall, the results of this study revealed the affecting path of social capital on subjective wellbeing in older people. Family, workplace, and community social capital were all major contributor and had direct effect on subjective wellbeing, and the mediating roles of family, workplace, community social capital between society social capital and subjective wellbeing were authenticated. The final model conduced a multi-level structure and emphasized the interactions between the elderly people with various social capital on micro (family), meso (workplace, community), and macro (society) levels and the relation to subjective wellbeing. It suggests that a rather strongly mutually dependent and interacting mechanism exists between the social capital and subjective wellbeing among the elderly.

The limitations of the study should be noted. Although structural equation models have advantages in establishing causality, the results cannot support the exact direction of causation because of the cross-sectional data. In this study, we revealed the direct effect of workplace social capital on subjective wellbeing among elderly people, future longitudinal studies should be considered to collected the workplace social capital of the elderly before and after retirement and verified the relationship. In addition, as social capital is generated in the interaction between mutual life environments, applicability of the affecting path revealed in our study should be verified in older groups from different districts.

## Conclusions

This study investigated the relationship between micro, meso, macro level social capital and subjective wellbeing of the elderly, special attention is paid to the role path of social capital at various levels. Results showed that family, workplace, community social capital directly affect the subjective wellbeing, Mediation analysis indicate that family, workplace, community social capital indirectly mediate the society social capital on subjective wellbeing. Social capital at all levels is the protective factor of the subjective wellbeing of the elderly. The subjective wellbeing of the elderly is related to their health status and quality of life. The health care system should not only focus on diseases and disabilities, but also to provide the supporting methods of the active psychological state. This study answers two key questions in a relatively comprehensive social capital structure, which social capital is useful for the subjective wellbeing of the elderly, and how the multi-level social capital works. This is important for us to understand the potential process of subjective wellbeing in positive psychology among older ages, and continue to invest in improving resources.

## Data Availability Statement

The raw data supporting the conclusions of this article will be made available by the authors, without undue reservation.

## Ethics Statement

The studies involving human participants were reviewed and approved by the Ethics Committee of the West China School of Public Health, Sichuan University. The patients/participants provided their written informed consent to participate in this study.

## Author Contributions

ZX, NL, and BG developed the concept and design. BG provided project administration and resources. ZX and WZ analyzed the data and wrote the manuscript. XZ and YW revised the manuscript. YW and QC conducted the survey. All authors read and approved the final manuscript.

## Funding

This research was jointly supported under the National Science Foundation of China (Grant No. 71603176).

## Conflict of Interest

The authors declare that the research was conducted in the absence of any commercial or financial relationships that could be construed as a potential conflict of interest.

## Publisher's Note

All claims expressed in this article are solely those of the authors and do not necessarily represent those of their affiliated organizations, or those of the publisher, the editors and the reviewers. Any product that may be evaluated in this article, or claim that may be made by its manufacturer, is not guaranteed or endorsed by the publisher.
